# Genetic Patterns in Familial Thoracic Aortic Aneurysm Disease

**DOI:** 10.3390/genes17101205

**Published:** 2026-09-29

**Authors:** Lisa C. Harling, Mohammad A. Zafar, Mah I. Kan Changez, Dimitra Papanikolaou, Nafiye Busra Celik, Nimrat Grewal, John A. Elefteriades

**Affiliations:** 1 Aortic Institute at Yale-New Haven, Yale University School of Medicine, Clinic Building CB 317, 789 Howard Avenue, New Haven, CT 06519, USA; lisa.harling@yale.edu (L.C.H.);; 2 Department of Cardiothoracic Surgery, Amsterdam University Medical Centres, 1081 HV Amsterdam, The Netherlands; 3 Department of Cardiothoracic Surgery, Leiden University Medical Center, 2333 ZA Leiden, The Netherlands

**Keywords:** aortic aneurysm, aortic dissection, aortic genetics, aortic surgery, genetic testing, whole exome sequencing, family patterns

## Abstract

Background and Aims: We examined familial patterns and phenotypes of thoracic aortic aneurysms and dissections (TAAD) in the 3746 patients currently populating our Yale Aortic Institute Database. Methods: Patient charts and electronic medical records were retrospectively reviewed for the entire cohort. TAAD disease was categorized as syndromic (with extra-aortic manifestations) or non-syndromic (without) for each patient. Family patterns and whole exome sequencing results were assessed. Family history was considered ‘proven’ when a patient’s relative(s) had an arterial aneurysm or dissection confirmed by an imaging study, surgical treatment, or postmortem examination. Family history was termed ‘likely’ when at least one relative suffered sudden death at ages ≤50 for males and ≤60 for females, and ‘possible’ with a history of sudden death at any age. Results: Of the 3746 patients in the database, family history of aneurysm or dissection and connective tissue status was able to be determined for 3113 patients, divided into 109 (3.5%) syndromic and 3004 (96.5%) non-syndromic TAAD patients. Findings were as follows: (1) In the syndromic group, 46 (42.2%) patients had a ‘proven’ family history, 6 (5.5%) had a ‘likely’ family history, 4 (3.7%) had a ‘possible’ family history, and 18 (16.5%) had ‘none’. For 35 patients (32.1%) their family history was unknown. (2) In the non-syndromic group, 587 (19.5%) patients had ‘proven’ family history, 147 (4.9%) had ‘likely’ family history, 210 (7.0%) had ‘possible’ family history and 1444 (48.1%) had no family history, thus representing sporadic TAAD. A total of 616 (20.5%) had an unknown family history. (3) There was a significant difference between the rate for ‘proven’ and ‘none’ family history between the syndromic and non-syndromic groups (42.2% vs. 19.5% and 16.5% vs. 48.1%, respectively; *p* < 0.001). (4) Syndromic patients presented at a significantly younger age than non-syndromic or sporadic patients (41.2 vs. 63.3 or 63.6; *p* < 0.001). (5) There was no significant difference in age of presentation between familial non-syndromic (patients with ‘proven’, ‘likely’, and ‘possible’ family history) and sporadic patients (63.3 vs. 63.6; *p* = 0.64). (6) Overall, of the 3746 patients in the database, 631 patients (25 syndromic, 552 non-syndromic, and 54 with unknown status) underwent Whole Exome Sequencing (WES). Sequencing revealed 170 disease-causing or suspicious variants [13 pathogenic, 16 likely pathogenic, 7 not classified, and 134 Variants of Uncertain Significance (VUS)] found in the exomes of 141 (22.3%) patients (12 syndromic, 103 non-syndromic TAAD patients plus a remaining 26 of unknown connective tissue status). Seventy (49.6%) of these patients had a ‘proven’ family history. (7) Familial TAAD patients were more likely to find a variant in their WES result than patients without a family history (70 vs. 26; *p* < 0.001). Recognition of these strong familial patterns, even in non-syndromic cases, encourages intensive investigation of family in order to save lives by detecting silent TAA disease. Conclusions: Our large-scale, clinical and sequencing-based study clearly demonstrates the strongly familial and genetically mediated nature of thoracic aortic disease. We vividly demonstrate and characterize this familial nature of TAAD, both clinically and by genetic sequencing. Our findings strongly support vigorous family investigation when a new patient is diagnosed with thoracic aortic aneurysm, as well as vigorous application of whole exome sequencing for these families.

## 1. Introduction

The heritable nature of aortic disease is currently well established. In abdominal aortic aneurysms, genetics and the role of family history have been known since the 1980s. However, it was not until the late 1990s that the familial nature of non-syndromic *thoracic* aortic disease was described [1,2,3,4]. Syndromic thoracic aortic aneurysm or dissection (TAAD) is associated with extra-aortic manifestations and caused by connective tissue disorders such as Marfan syndrome, Ehlers–Danlos syndrome, Loeys-Dietz syndrome, and others [5]. Syndromic TAAD accounts for less than 5% of all TAAD cases, leaving many other aneurysm families unexplained [6,7].

The initial reports of familial aggregation of TAAD by Milewicz and by our group observed a 21% incidence of familial thoracic aortic aneurysm and, most frequently, an autosomal dominant mode of inheritance [3,4,8]. Since then, genetic variants in at least 70 genes have been associated with TAAD [9,10]. The current guidelines for the diagnosis and management of patients with thoracic aortic disease recommend aortic imaging for first-degree relatives of patients with thoracic aortic aneurysm (TAA) and/or dissection to identify those with asymptomatic disease (Class 1, Level B), and aortic imaging for second-degree relatives if one or more first-degree relatives are found to have thoracic aortic dilatation, aneurysm, or dissection (Class 2, Level B) [11].

As molecular and genetic understanding of thoracic aortic disease advances, it becomes even more important to examine clinical patterns of familial clustering of TAAD. The primary objective of the present study was to confirm the familial nature and frequency of TAAD in a large population of affected patients and to characterize familial clustering and phenotype patterns in the same population. We also sought to explore the precise genetic findings in a sizeable subset of patients who underwent whole exome sequencing (WES).

## 2. Methods

This study was approved by the Yale University Human Investigation Committee (12617).

### 2.1. Patients

Currently, there are 3746 patients in the Database of the Aortic Institute at Yale-New Haven Hospital, diagnosed with TAA and/or TAAD. These individuals comprise the patient population for the current study. Patient charts and electronic medical records were retrospectively reviewed for all patients. A patient was considered to have a connective tissue status if confirmed by genetic testing or if manifesting classic clinical stigmata of Marfan, Ehlers–Danlos, or Loeys-Dietz syndromes, as judged by the senior author (J.A.E).

### 2.2. Family History

Family history data were extracted from detailed, in-person patient interview records written by the senior author (J.A.E). Every patient was asked the questions: (1) Have any of your family members had an aneurysm or dissection? (2) Have there been any sudden deaths in your family? Family history was considered as ‘proven’ only for patients with ≥ 1 relative(s) with an arterial aneurysm or dissection of any location, confirmed by an imaging study, surgical treatment, or postmortem examination. Family history was classified as ‘likely’ for patients with ≥1 relative(s) who suffered sudden death at ages ≤50 for males and ≤60 for females, and ‘possible’ with a history of sudden death at any age. Furthermore, family history was categorized as ‘none’ when none of the patient’s relatives suffered documented TAAD or sudden death, and as ‘unknown’ whenever data were insufficient to determine family history.

### 2.3. Genetic Testing

The Aortic Institute’s database predates ubiquitous availability of clinical genetic testing [11]. Genetic testing at the Aortic Institute commenced in 2012 with a grant and nowadays is offered to all the aneurysm patients at the Aortic Institute, employing WES as part of our routine testing program [12,13]. Unfortunately even today many patients encounter prohibitive conditions, as many insurance companies do not approve the coverage of WES testing and sequencing remains costly enough that most families are unable to afford it.

Saliva or blood samples were obtained, DNA was extracted, and the entire exome was sequenced, searching for genetic variants potentially causing TAAD. The most recent TAAD gene set analyzed via WES at our laboratory includes the following 70 genes: *ABCC6*, *ABL1*, *ACTA2*, *ACVR1*, *ADAMTS10*, *ALDH18A1*, *ARIH1*, *ASPH*, *ATP6V0A2*, *ATP7A*, *B4GALT7*, *BGN*, *CBS*, *CHST14*, *COL1A1*, *COL1A2*, *COL2A1*, *COL3A1*, *COL4A1*, *COL5A1*, *COL5A2*, *COL9A1*, *COL9A2*, *COL9A3*, *COL11A1*, *COL11A2*, *EFEMP2*, *ELN*, *EMILIN1*, *FBLN5*, *FBN1*, *FBN2*, *FKBP14*, *FLCN*, *FLNA*, *FOXE3*, *HEY2*, *HNRNPK*, *IPO8*, *KCNN1*, *LOX*, *LTBP2*, *LTBP3*, *MAT2A*, *MED12*, *MFAP5*, *MYH11*, *MYLK*, *MYLK2*, *NOTCH1*, *PKD1*, *PKD2*, *PLOD1*, *PMEPA1*, *PRKG1*, *SECISBP2*, *SK1*, *SLC2A10*, *SCL39A13*, *SMAD2*, *SMAD3*, *SMAD4*, *SMAD6*, *TGFB2*, *TGFB3*, *TGFBR1*, *TGFBR2*, *THSD4*, *TNXB*, *ZNF469* [10]. Variants found in one or more of the TAAD-associated genes were classified (by the geneticist) according to the criteria of the American College of Medical Genetics and Genomics (ACMG) as pathogenic, likely pathogenic, benign, likely benign, or variants of uncertain significance (VUS) [14].

### 2.4. Statistics

Statistical analysis was performed using R 4.4.2 (R Foundation for Statistical Computing, Vienna, Austria). Data are expressed as mean ± standard deviation for continuous variables and as numbers (percentages) for categorical variables. A two-level approach, firstly utilizing a Kruskal–Wallis omnibus test to search for any occurrence of statistical difference, followed by a Dunn’s test, with a Benjamini–Hochberg (FDR) correction to account for multiple comparisons, was used to compare the mean age at presentation in different patient subgroups. The employment of the non-parametric test accounts for the skewed age-distribution in the groups (Supplemental Figure S1), as well as the high variation in sample size. A Pearson’s chi-square test was utilized to find statistical differences in the positivity rate for family history between non-syndromic and syndromic patients. Additionally, a Pearsons chi-square test (Fisher’s Exact test for values < 5) was utilized to compare the differences between comorbidities and ‘guilty associations’ (between the three groups: syndromic, familial non-syndromic, and sporadic). (“Guilty associations” refers to the presence of the number of lesions in other organs known to be associated with thoracic aortic disease. These include intracranial aneurysm, abdominal aortic aneurysm, renal cyst, temporal arteritis, bovine aortic arch, bicuspid aortic valve, positive thumb-palm sign, and positive family history) [15].

## 3. Results

### 3.1. Patient Characteristics

From the total of 3746 patients in the Aortic Institute database at Yale-New Haven Hospital, family history and connective tissue status were determined for 3113 patients. The remaining 633 patients had unknown parameters for family history or connective tissue status. The 3113 patients were subdivided into 3004 (96.5%) non-syndromic and 109 (3.5%) syndromic TAAD patients. The syndromic group included six patients with positive WES for Marfan syndrome, three for Loeys-Dietz syndrome, and one for Ehlers–Danlos syndrome. The remaining 99 patients in the syndromic group either did not undergo genetic testing, or genetic variants were found in the gene testing but they additionally exhibited extra-aortic manifestations or had other syndromic genes. From our patient cohort, 324 (10.4%) suffered a Type A dissection, 271 (8.7%) suffered a Type B dissection, and 99 (3.2%) presented with rupture. 644 (20.7%) patients had bicuspid aortic valves on imaging. Patient characteristics are shown in Table 1.

### 3.2. Non-Syndromic vs. Syndromic TAAD

**Figure 1:**
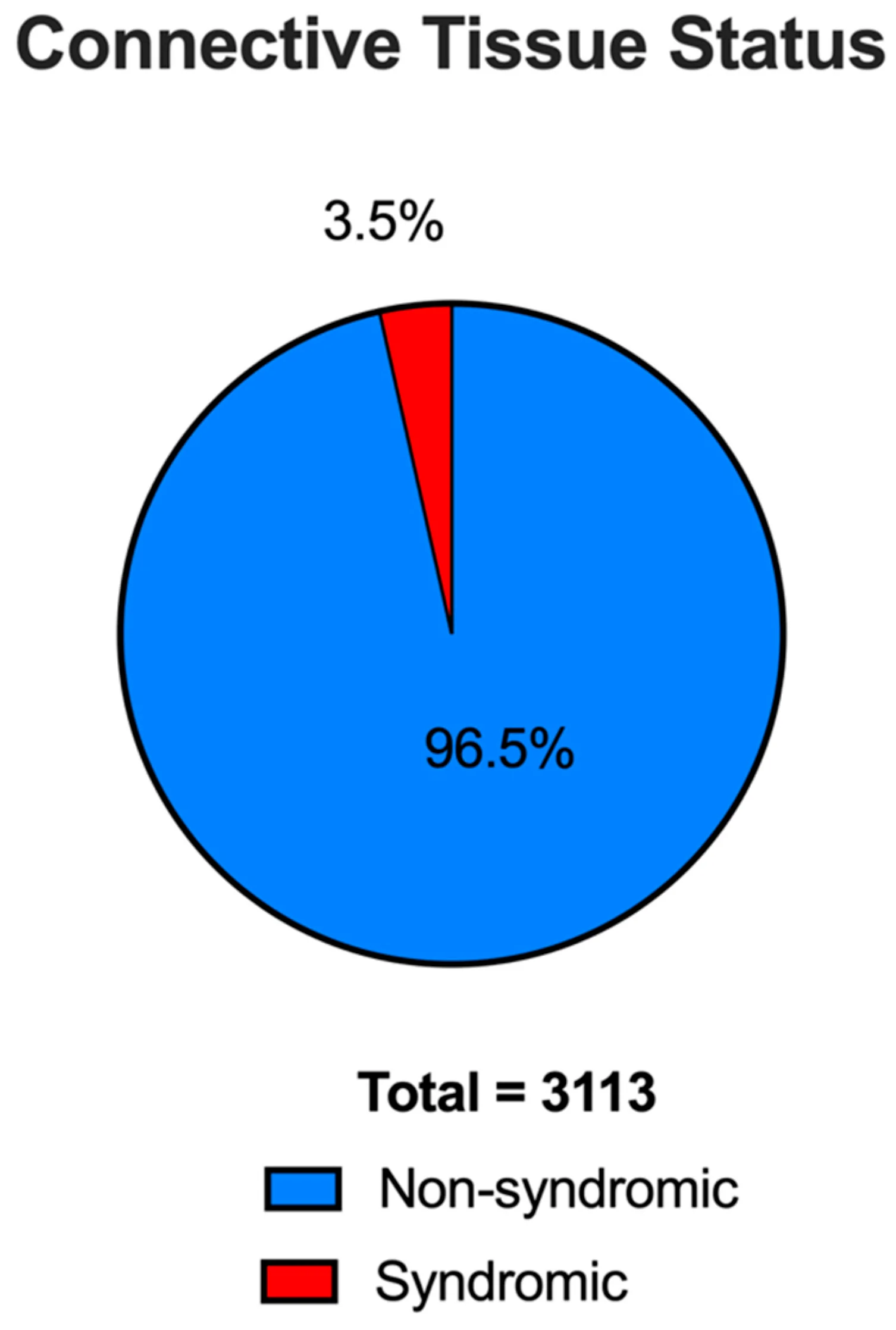
Distribution of non-syndromic and syndromic TAAD patients in the patient cohort.

**Figure 2:**
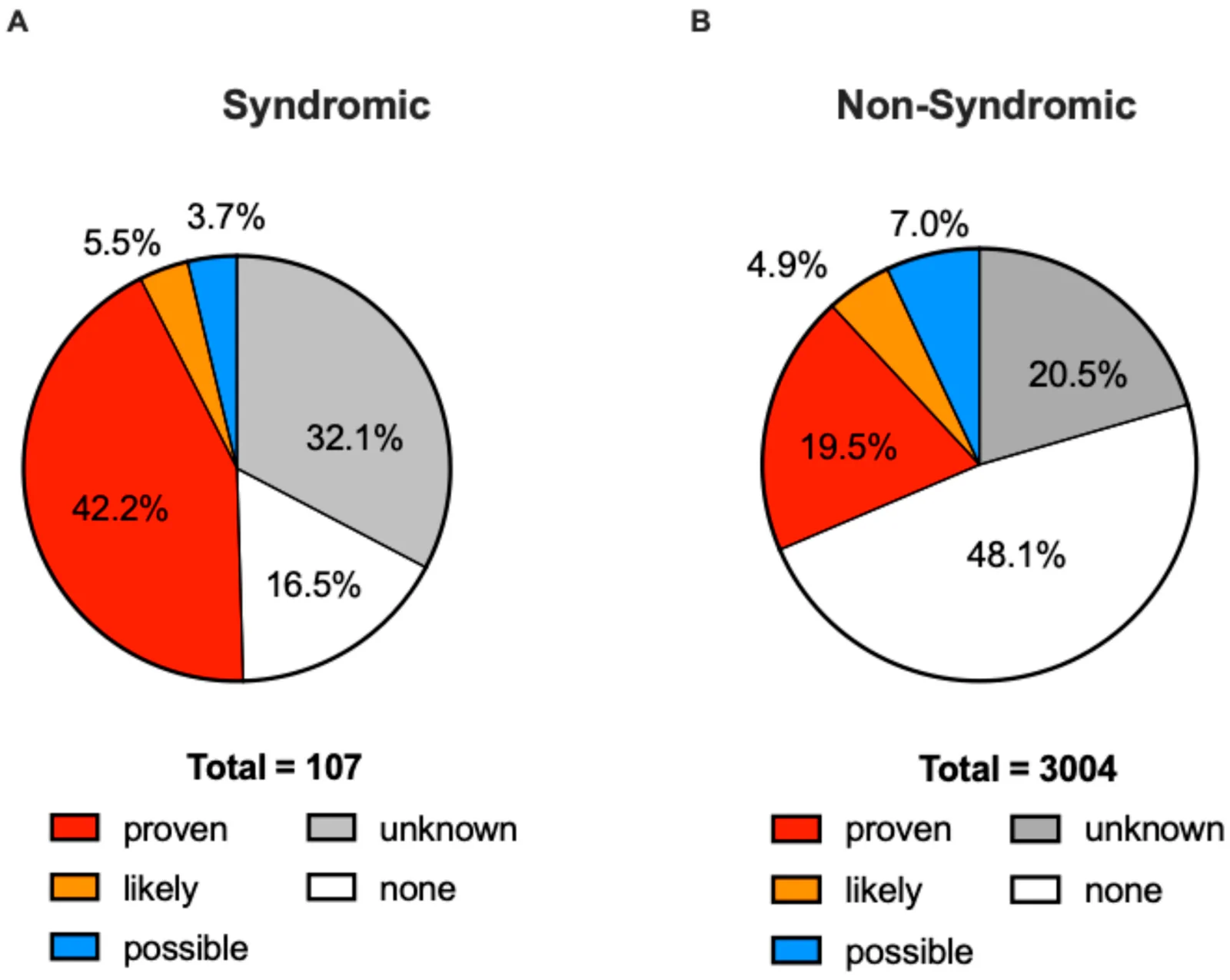
(**A**) Distribution of positive family history in Non-Syndromic TAAD patients subdivided into ‘proven,’ ‘likely’, ‘possible’, ‘unknown’, and ‘none’. (**B**) Distribution of positive family history in Syndromic TAAD patients subdivided into ‘proven’, ‘likely’, ‘possible’, ‘unknown’, and ‘none’.

Overall, family history was proven in 587 patients in the non-syndromic and 46 in the syndromic group (Figure 1). In the non-syndromic group, 587 (19.5%) patients had a ‘proven’ family history of aneurysm or dissection (representing familial non-syndromic TAAD). Additionally, in the non-syndromic group, family history was classified as ‘likely’ for 147 (4.9%), ‘possible’ for 210 (7.0%), ‘none’ for 1444 (48.1%) (representing truly sporadic TAAD), and for 616 (20.5%) their family history was unknown (Figure 2A). In the syndromic group, 46 (42.2%) patients had a ‘proven’ family history, six (5.5%) had a ‘likely’ family history, four (3.7%) had a ‘possible’ family history, 18 (16.5%) had ‘none’, while for 35 (32.1%) their family history was unknown (Figure 2B). There was a significant difference between the positivity rate for ‘proven’ and ‘none’ family history between the syndromic and non-syndromic groups (42.2% vs. 19.5% and 16.5% vs. 48.1%, respectively; *p* < 0.001). Thirty-five patients in the syndromic group (32.1%) presented with acute aortic dissection, while 510 patients in the non-syndromic group (17.0%) presented with acute aortic dissection. The syndromic group presented at a younger age than non-syndromic TAAD patients (41.2 vs. 63.3; *p* < 0.001). Familial non-syndromic patients presented at a similar age to sporadic TAAD patients (63.3 vs. 63.6; *p* = 0.64). Patient characteristics are presented in Table 1 and Table 2.

### 3.3. Comorbidities and Other Medical Conditions Associated with TAA

The details of the associated comorbidities and medical conditions have been summarized in Table 3. Statistical significance was noted for the following medical condition: bicuspid aortic valve (BAV), with a higher incidence observed in non-syndromic and sporadic TAAD patients compared to syndromic cases.

### 3.4. Whole Exome Sequencing

**Figure 3:**
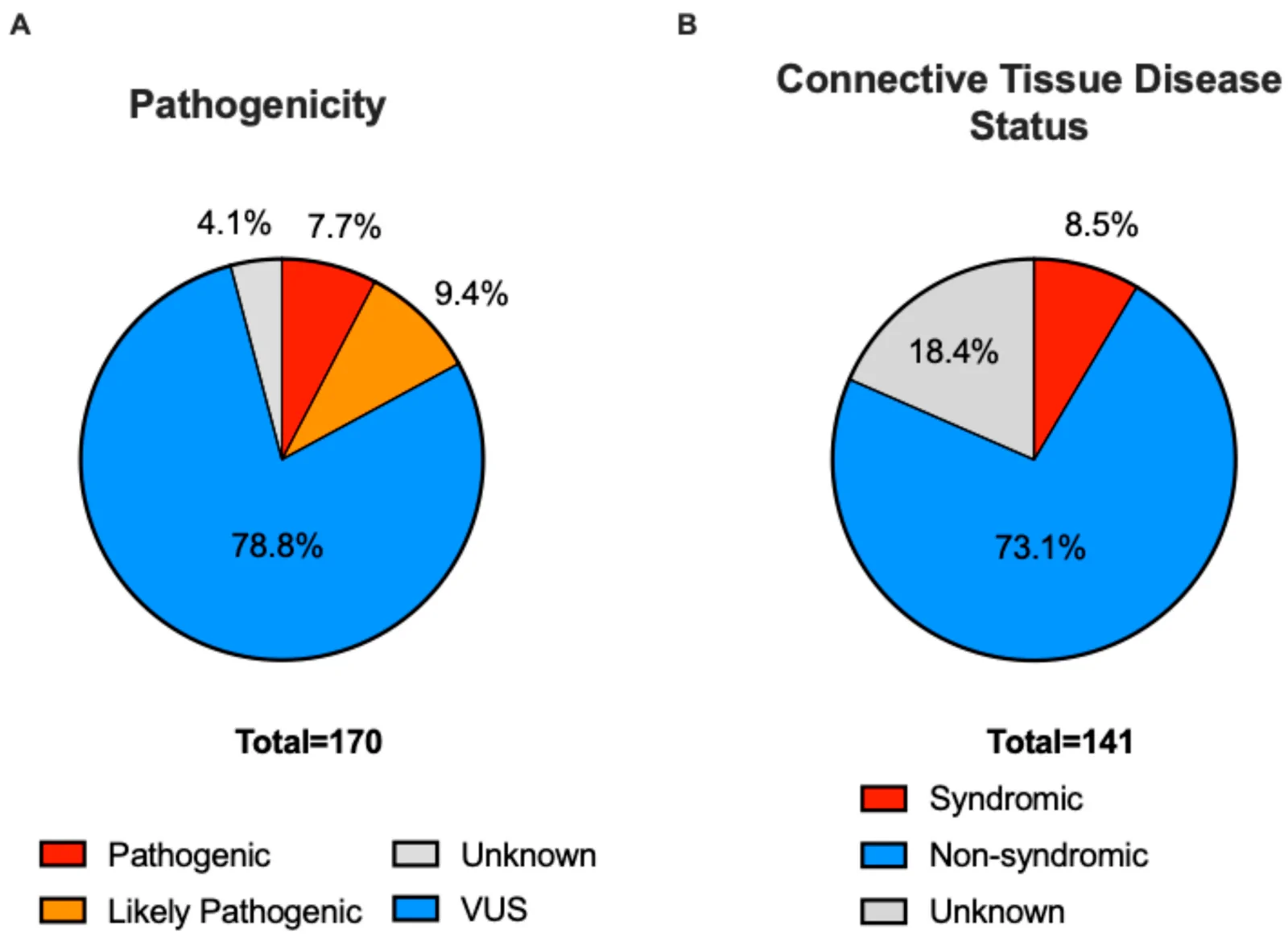
(**A**) Distribution of positive WES testing in patients subdivided into ‘pathogenic,’ ‘likely pathogenic’, ‘Variant of Uncertain Significance’, and ‘unclassified’. (**B**) Distribution of positive WES testing in patients subdivided into the status of connective tissue disease (‘syndromic,’ ‘non-syndromic’, ‘unknown’). VUS: variant of uncertain significance.

From the Aortic Institute’s database, 631 (16.8%) patients (25 syndromic patients, 552 non-syndromic patients, and 54 patients with unknown syndromic status) underwent genetic testing utilizing WES (Table 4). Of the patients who underwent WES, 179 (28.4%) had a ‘proven’ family history, and 263 (41.7%) had no family history (Figure 3A). WES resulted in a positive finding in 141 patients, meaning at least one variant was found in the patients’ genome (Table 4). WES revealed variants in 70 (23.9%) familial TAAD patients (proven family history). In these 70, at least one pathogenic, likely pathogenic variant, or variant of uncertain significance (VUS) was found. Familial TAAD patients were more likely to have a positive WES result than patients without a family history (‘none’) (70 vs. 26; *p* < 0.001, Table 5). Patients with non-syndromic TAAD were more likely to have a positive WES result than patients with syndromic TAAD (103 vs. 12; *p* < 0.001), which is counterintuitive given the hereditary nature of syndromic TAAD (Figure 3B). About half of patients with positive WES testing were positive for BAV (51.8%) (Table 4). Patients with a BAV were more likely to have received a negative than a positive WES result (72 vs. 28; *p* < 0.001), likewise counterintuitive given the hereditary nature of BAV [16]. Fourteen patients that presented with acute aortic dissection had a positive WES result (40.0% of acute aortic dissection patients that received WES testing), while 21 (60.0%) had a negative result (Table 4).

**Figure 4:**
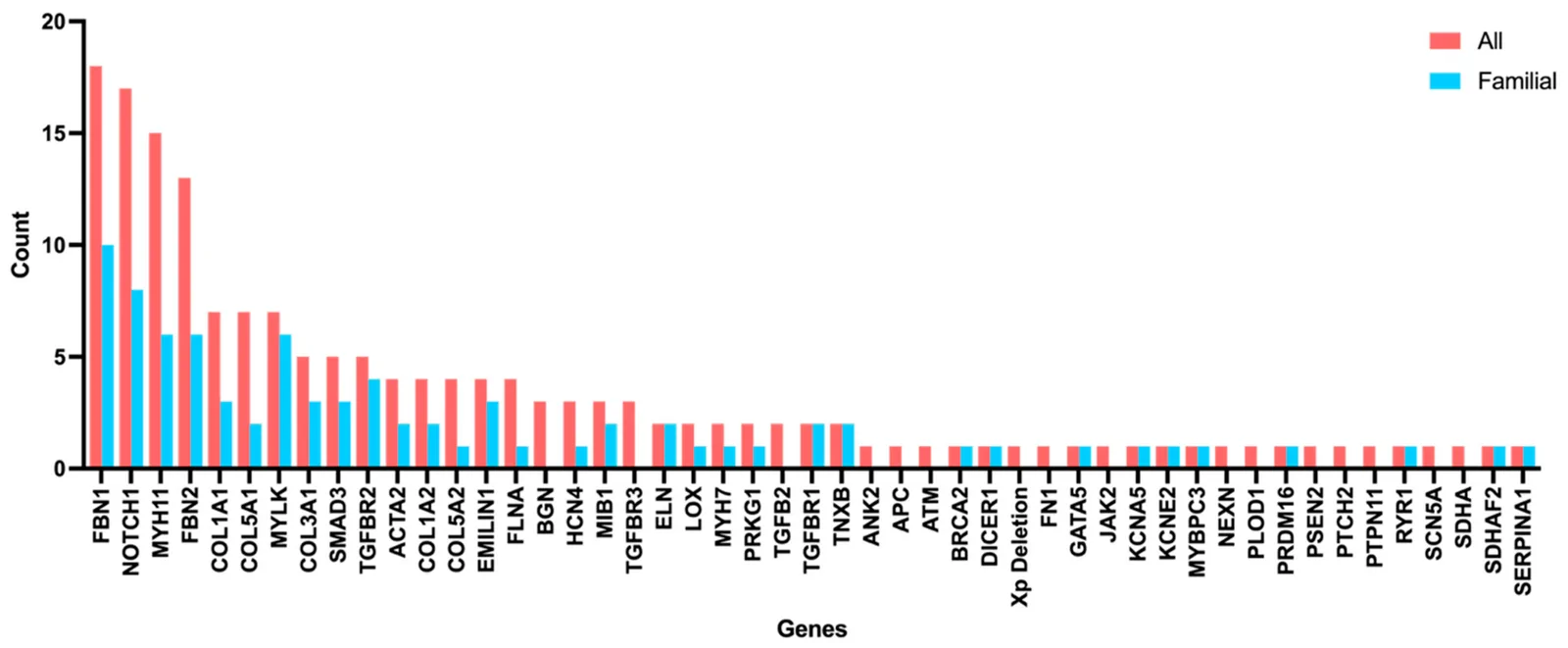
Distribution of genetic variants in TAAD-related genes among all patients with positive WES (*n* = 631, 170 genetic variants) and among the patients with positive WES and a proven family history of aneurysm/dissection (*n* = 70, 83 genetic variants). Blue columns represent the number of specific genes found in patients with familial TAAD, red columns represent the number of specific genes found in all tested TAAD patients (including familial TAAD cases).

In total, 170 genetic variants were discovered in the exomes of 141 patients with positive WES and varying family history. Some patients carried more than one variant in the same or different genes, some variants were found in the same form in multiple patients, such as in *NOTCH1*, *MIB1*, *MYH11*, and *TGFBR1*, leading to 164 different genetic variants. Of these 170 variants, 13 (7.6%) were classified as pathogenic, 16 (9.4%) likely pathogenic, and 134 (78.8%) as VUS, while 7 (4.1%) were not classified (Table 5). TAAD-related genetic variants were prevalent in the *FBN1* gene (*n* = 18, 10.6%) and *NOTCH1* (*n* = 17, 10.0%), followed by the *MYH11* gene (*n* = 15, 8.8%), and *FBN2* (*n* = 13, 7.6%). Among 83 patients with a proven family history, harbored variants included *FBN1* (*n* = 10, 12.0%), *NOTCH1* (*n* = 8, 9.6%), *FBN2* (*n* = 6, 7.2%), *MYH11* (*n* = 6, 7.2%), and *MYLK* (*n* = 6, 7.2%). Among the 14 patients with a positive WES result that had presented with acute aortic dissection, 17 variants were found and included *FBN1* (*n* = 6, 35.3%), *NOTCH1* (*n* = 1, 5.9%), *MYLK* (*n* = 2, 11.8%), *ACTA2* (*n* = 2, 11.8%), and *COL1A1* (*n* = 1,5.9%). Figure 4 shows the distribution of the genetic variants in TAAD-related genes among the patients with positive WES, as well as among the patients with positive WES and proven family history of aneurysm/dissection.

## 4. Discussion

This study looks at the familial and genetic features of a large, single center group of thoracic aortic aneurysm patients cared for over several decades at a single institution. This provides the opportunity to “gauge the nature of the beast”, as the expression goes.

The findings of the present study provide deeper support for our prior preliminary demonstrations of the familial nature of non-syndromic thoracic aortic disease. We found that 19.5–31.4% of non-syndromic TAAD patients in our large cohort had a documented family history of arterial aneurysm or dissection (Table 1). This result expands upon earlier groundbreaking work by Milewicz and colleagues, as well as prior studies by our group in smaller patient sets from two decades ago, which had reported that 21% of thoracic aortic aneurysms are familial [3,4,5,6,7,8]. However, the true incidence of familial thoracic aortic disease is likely underestimated by these studies. It is possible that relatives of patients categorized as ‘sporadic’ harbor undiagnosed, silent aneurysms without having ever been imaged. Additionally, as thoracic aortic aneurysm is most commonly a disease of middle age, younger relatives of ‘sporadic’ TAAD patients might have not yet developed an aneurysm or dissection at the time a specific imaging study was conducted. Recent work by Raunso et al. found that first-order relatives of TAAD patients experience a 9-fold risk-increased rate of having an aortic aneurysm or dissection compared to the general population [17]. In a previous study, our group reported that the probability of aortic dissection was 2.77 times higher in TAA patients with a positive family history than in patients with a negative family history of aortic dissection [18].

A higher prevalence of bicuspid aortic valves (BAV) was observed in the non-syndromic and sporadic groups, compared to the syndromic group. Additionally, BAV patients had a significantly higher rate of negative WES results than positive (72 vs. 28; *p* < 0.001). BAV is a disease of heritable nature and has partially been linked to genes also involved in syndromic TAAD, such as *FBN1* or *TGFBR2* [16]. In our cohort of BAV patients, two (2% of tested BAV patients) were found to have variants in the *FBN1* gene. Other genetic variants linked to BAV include *NOTCH1* (6%), *GATA6* (0%), *ACTA2* (0%), *HOXA1* (0%), and *KCNJ2* (0%) [16]. Percentages are related to the number of BAV patients in our cohort with variants in the specific gene. The overall high prevalence of BAV in the cohort may be a result of common referral patterns, as aortopathy in BAV patients may be detected during routine examinations of the aortic valve.

No other comorbidities resulted in significant differences between groups. However, the syndromic group showed a trend towards a lower prevalence of hypertension, dyslipidemia, diabetes mellitus, myocardial infaction, coronary artery disease, smoking, bovine and other arch abnormalities, history of steroid use, and chronic kidney disease, while the prevalence in the non-syndromic and sporadic group showed similar results. A trend towards a higher prevalence in the syndromic group was noted with coarctation of the aorta, AAAs, renal and liver cysts (Table 3). These reults, despite not being significant, underscore that the clinical information on family history of aneurysm or dissection is especially valuable when supplemented by comprehensive genetic testing of probands. Upon discovery of a definite or probable disease-causing variant, WES of family members can be carried out, therby identifying other individuals at risk and suscptible to aortic events. The end result of such an approach can be expected to be saved lives.

We performed WES in 631 patients in this cohort, of whom 141 (22.3%) were found to have genetic variants (Table 4). VUS were categorized as suspicious based on characteristics such as rarity across population databases (variants with a high population frequency have a low probability of pathogenicity, whereas extremely rare variants are more likely to be disease-causing), conservation of affected alleles across species in phylogeny, and prediction of variant pathogenicity by in silico tools. Currently, with at least 70 TAAD-associated genes discovered, WES yields positive results in about one-third of TAAD patients [10]. In our cohort, 70 of 179 sequenced familial TAAD patients had positive WES findings (39.1%). The genes most commonly affected overall were the *FBN1* gene (*n* = 18, 10.6%) and *NOTCH1* (*n* = 17, 10.0% ), followed by the *MYH11* gene (*n* = 15, 8.8%), and *FBN2* (*n* = 13, 7.6%). *FBN1* is mainly associated with Marfan syndrome, while *NOTCH1* and *MYH11* are known to be associated with familial non-syndromic TAAD and aortic dissection at small diameters. *FBN2* has been linked to other forms of syndromatic TAAD [9,19].

**Figure 5:**
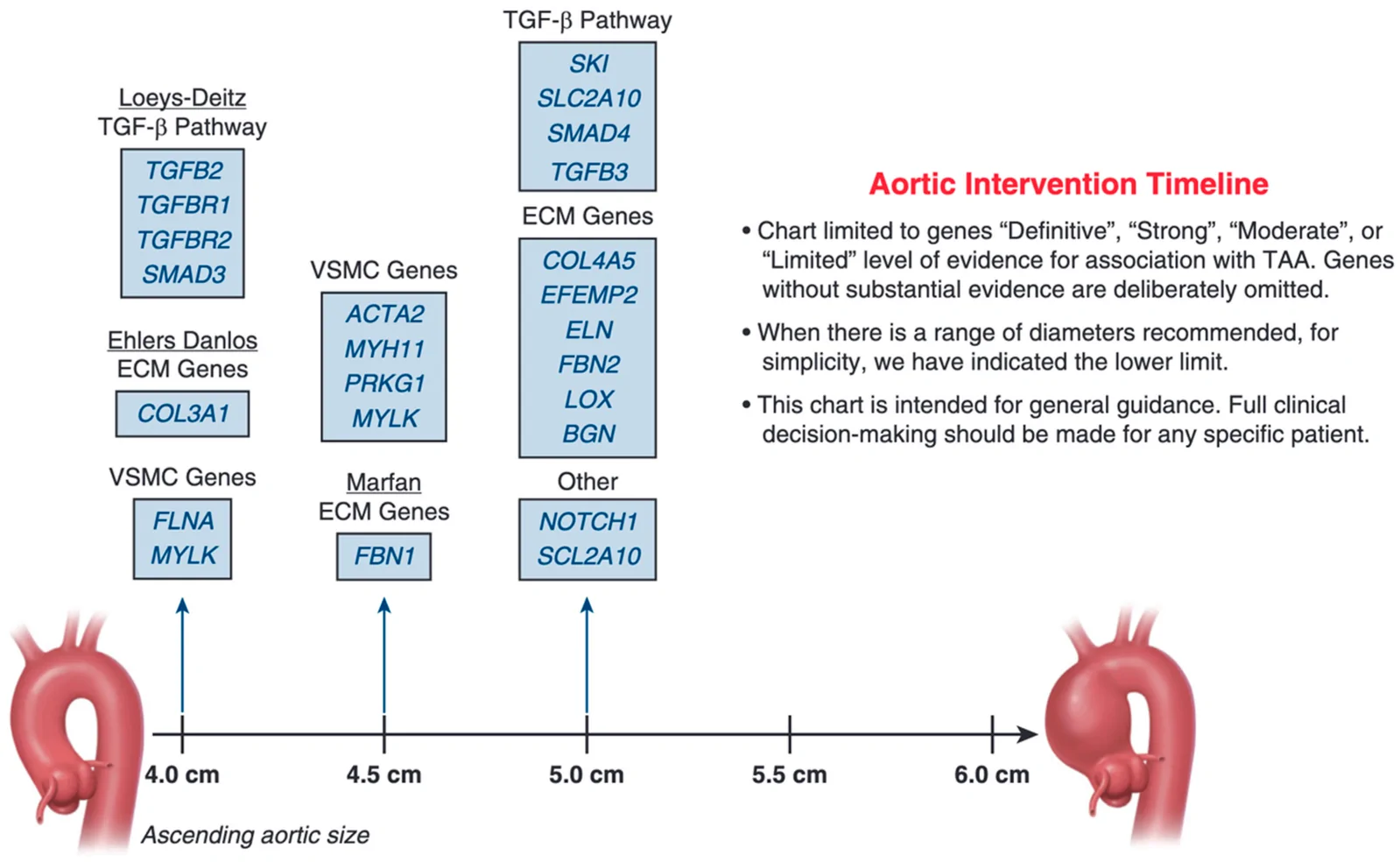
Ascending aorta dimensions for prophylactic surgical intervention. Abbreviations: ECM: extracellular matrix; TAA: thoracic aortic aneurysm; TGF: transforming growth factor; VSMC: vascular smooth muscle cell. (Reproduced with permission from Elefteriades JA, Zafar MA, Ziganshin BA. Genetics of aortic aneurysm disease: 10 key points for the practitioner. *JTCVS Open*. 2024;21:58–63. doi:10.1016/j.xjon.2024.07.014 [10]).

Knowledge of the combination of family patterns and the underlying genetic variant in individual TAAD patients holds direct clinical implications. For the ascending aorta, size cutoffs for aneurysm resection have been proposed according to the affected gene (Figure 5) [20]. This approach heralds truly personalized, molecular-based care. Personalized medicine in general has greatly improved in recent years due to improved technologies and has led to advances in diagnostic accuracy and customized care [21]. Genomic and metabolic markers are the main targets of this personalized approach and can be utilized to customize pharmacological strategies [21]. Single-site Sanger sequencing (simpler and cheaper than WES) for the variant identified in the proband permits the identification of genetically at-risk individuals in the family. Discovery of undiagnosed aneurysms can lead to surgical intervention at the appropriate size threshold. Elective ascending aortic surgery demonstrates more than 98% safety in specialized centers and is the only method for preventing cataclysmic TAAD complications such as acute dissections and sudden death [9,20,22,23].

This study vividly demonstrates the familial nature of TAAD, both clinically and by genetic sequencing. By pursuing rigorous family screening and supplementing with gene analysis by WES, many unsuspecting family members who silently carry TAAD disease may be identified. Thereafter, careful clinical observation and appropriately timed surgery will save their lives.

## 5. Limitations

**Figure 6:**
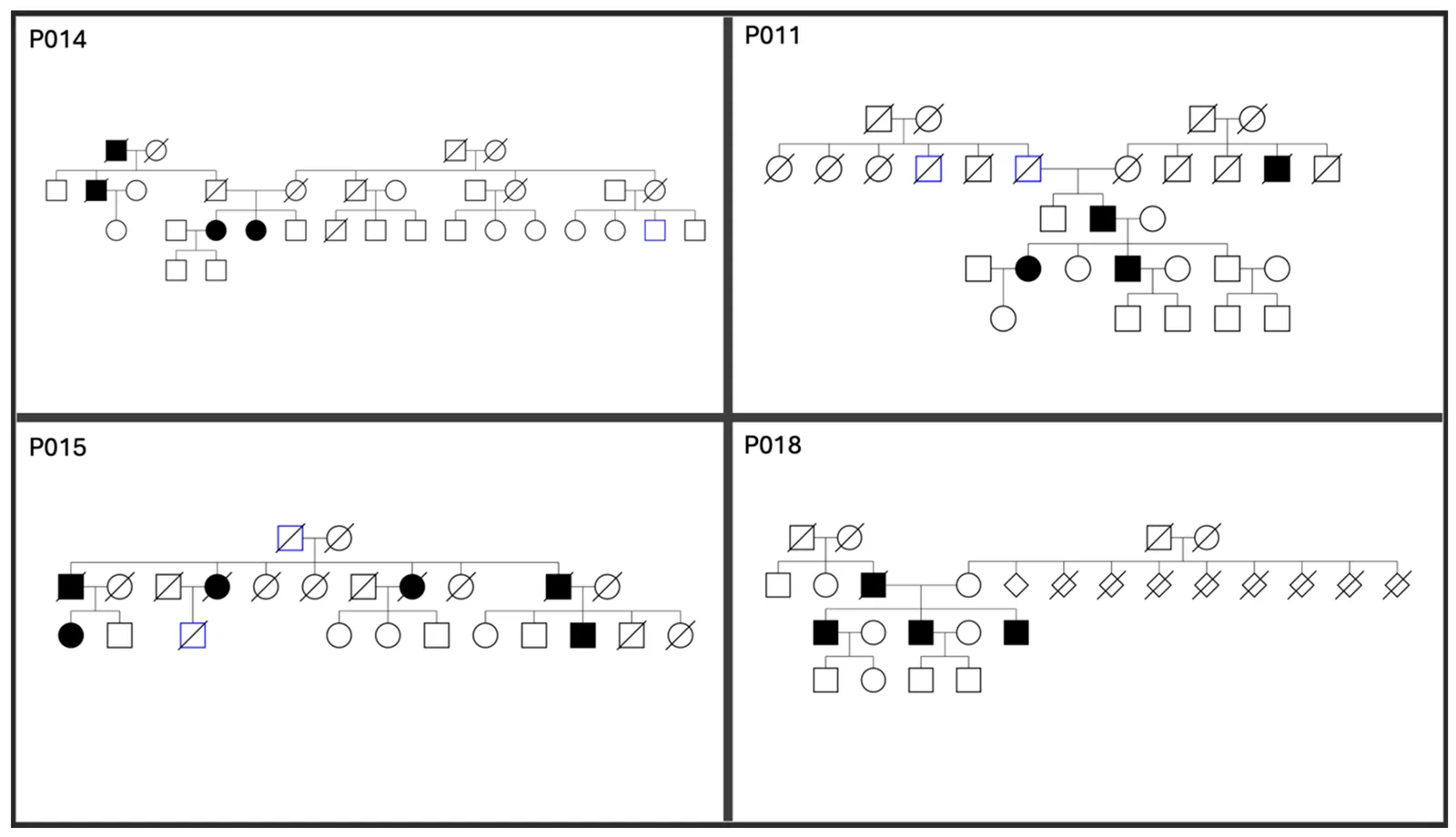
Non-syndromic thoracic aortic aneurysm family pedigrees. Squares represent men, circles represent women, and diamond shapes represent unknown gender. Blackened squares or circles represent affected patients with aortic aneurysms, blue squares or circles represent unknown disease status. Crossed out squares or circles represent deceased individuals. Visualization of pedigrees was done using QuickPed 4.5.0 (Oslo, Norway) [24].

This report is based on general family histories obtained during clinical intake of new patients presenting to our Aortic Institute by the senior investigator, not on complete family pedigrees. Selected pedigrees from families with affected individuals in multiple generations are included (Figure 6 and Supplemental Figure S2). However, our standard questions (Do you have family members with aneurysm disease? Has any relative suffered sudden death?) are quite powerful for detection of familial thoracic aortic disease. Focused studies with formal, complete pedigrees could be more revealing. However, if our two standard questions are answered negatively, it would take aortic imaging or genetic sequencing to uncover additional affected family members. This study is also dependent on accurate patient reports of affected individuals in their families. True incidences likely would be considerably higher, were family members screened specifically for the purposes of such a study.

Nonetheless, despite “real-world” limitations, this report on a large group of affected patients provides an informative view of the general genetic clinical contours of thoracic aortic aneurysm disease.

## 6. Conclusions

We present this study of a very large number of TAA patients (ascending and descending) whose family histories were determined over decades at our Aortic Institute at Yale University. Our study reaffirms (in this large patient sample) that thoracic aortic aneurysms and dissections demonstrate strong familial clustering—not only in syndromic families, but also in at least 24.6% of non-syndromic cases.

This recognition sets the stage for saving lives by encouraging vigorous investigation of at least all first-order family members of probands of patients with TAA.

Specific key messages regarding family patterns in TAAD are as follows:

(1) High likelihood of positive family history in the syndromic group. In the syndromic group, 46 (42.2%) patients had a ‘proven’ family history, 6 (5.5%) had a ‘likely’ family history, 4 (3.7%) had a ‘possible’ family history, and 18 (16.5%) had ‘none’ (Table 1).
(2) Substantial likelihood of positive family history even in the non-syndromic group. In the non-syndromic group, 587 (19.5%) patients had ‘proven’ family history, 147 (4.9%) had ‘likely’ family history, 210 (7.0%) had ‘possible’ family history, and 1444 (48.1%) had no family history, representing sporadic thoracic aortic aneurysm and dissection (TAAD) patients.
(3) Syndromic patients were more likely to manifest a positive family history. There was a significant difference between the positivity rate for ‘proven’ family history between the syndromic and non-syndromic groups (42.2% vs. 19.5%; *p* < 0.001).
(4) Syndromic patients present at an earlier age. Syndromic patients presented at a significantly younger age than non-syndromic patients (41.2 years vs. 63.3 years; *p* < 0.001).
(5) There was no significant difference in age of presentation between familial non-syndromic (patients with ‘proven’, ‘likely’, and ‘possible’ family history) and sporadic patients (63.3 vs. 63.6; *p* = 0.64).
(6) Whole exome sequencing is revealing in both syndromic and non-syndromic scenarios. Upon genetic sequencing from a total of 631 patients, 170 genetic variants (134 (78.8%) variants of uncertain significance (VUS)) were found in 141 (22.3%) patients (12 syndromic, 103 non-syndromic TAAD patients, 26 with unknown connective tissue status).
(7) Familial patients have higher WES return. Familial TAAD patients were more likely to have a positive WES result than patients without a family history (70 vs. 26; *p* < 0.001).

The real frequency of hereditary TAAD is certainly even higher, as many family members (most, in fact) in the general population have never undergone aortic imaging. Image screening of (at least) all first-order family members of even non-syndromic patients is recommended. Investigation by genetic testing (WES for the proband, at least single-site Sanger sequencing for family members) is strongly recommended.

In the future, we anticipate that advances in clinical genetics and the proliferation of WES for thoracic aortic disease will permit the identification of many additional causative genetic variants. Personalized genetic understanding will enhance timely, preemptive surgical intervention and diminish the catastrophic complications of this virulent killer.

## Figures and Tables

**Table 1:** Patient demographics and clinical characteristics for the entire cohort (*n* = 3113). Headings and subheadings are in bold. Statistical analysis utilizing Dunn’s test, with a Benjamini–Hochberg (FDR) correction for the age at presentation. Significant *p*-value < 0.001 (*). ^1^ Data regarding occurrence of Type A or B dissection was not available for 2518 patients.

Variables	*n* (%) or Mean ± Standard Deviation
Total number of patients	3113
Females	936 (30.1%)
Bicuspid Aortic Valve	644 (20.7%)
**Category**	
Syndromic TAAD	109 (3.67%)
Non-syndromic TAAD	3004 (96.3%)
Sporadic	1444 (48.1%)
**Aortic Dissections (Acute or Chronic) or Rupture**	
Type A Dissection ^1^	324 (10.4%)
Type B Dissection ^1^	271 (8.7.0%)
Rupture	99 (3.2%)
Acute Aortic Dissection	546 (17.5%)
Syndromic	35 (6.6%)
Non-syndromic	510 (93.4%)
**Age at Presentation (Years)**	
Overall	62.6 ± 14.5
Syndromic TAAD	41.2 ± 16.6 *
Non-syndromic TAAD	63.3 ± 13.8
Sporadic	63.6 ± 14.1
**Syndromic Family History**	
Proven	46 (42.2%) *
Likely	6 (5.5%)
Possible	4 (3.7%)
None	18 (16.5%) *
Unknown	35 (32.1%)
**Non-syndromic Family History**	
Proven	587 (19.5%) *
Likely	147 (4.9%)
Possible	210 (7.0%)
None	1444 (48.1%) *
Unknown	616 (20.5%)
**Acute Aortic Dissection Family History**	
Proven	64 (11.7%)
Likely	10 (1.8%)
Possible	16 (2.9%)
None	284 (52.0%)
Unknown	172 (31.5%)

**Table 2:** Condensed genetic characteristics of 3113 thoracic aortic aneurysm patients (clinical). Statistical analysis utilizing two-tailed, unpaired Student’s *t*-test for the age at presentation. Significant *p*-value < 0.001 (*).

*n* = 3113	Proven	Likely	Possible	None	Unknown	AGE (yrs) (Mean)
Syndromic Group 109 (3.5%)	46 (42.2%) *	6 (5.5%)	4 (3.7%)	18 (16.5%) *	35 (32.1%)	41.2 ± 16.6 *
Non-syndromic Group 3004 (96.5%)	587 (19.5%) *	147 (4.9%)	210 (7%)	1444 (48.1%) *	616 (20.5%)	63.3 ± 13.8 *

**Table 3:** Comorbidities and other medical conditions associated with syndromic, non-syndromic, and sporadic TAA. Statistical analysis utilizing Pearson’s chi-square test; for values < 5, Fisher’s exact test was used. Significant *p*-value < 0.05 (*). The comorbidity profile is remarkably similar between syndromic and fanilial non-syndromic groups.

Comorbidities	Syndromic (*n* = 109)	Non-Syndromic (*n* = 3004)	Sporadic (*n* = 1444)	*p* Value
Hypertension	43 (39.4%)	1415 (47.1%)	681 (47.2%)	0.284
Dyslipidemia	21 (19.3%)	858 (28.6%)	393 (27.2%)	0.081
Diabetes Mellitus	3 (2.8%)	180 (6.0%)	90 (6.2%)	0.373
Myocardial Infarction	2 (1.8%)	75 (2.5%)	39 (2.7%)	0.887
Coronary Artery Disease	9 (8.3%)	503 (16.7%)	240 (16.6%)	0.063
Smoking	27 (24.8%)	945 (31.5%)	451 (31.2%)	0.335
Bicuspid Aortic Valve	12 (11.0%)	632 (21.0%)	335 (23.2%)	0.007 *
Bovine Arch	11 (10.1%)	376 (12.5%)	180 (12.5%)	0.753
Other Arch Anomalies	2 (1.8%)	65 (2.2%)	36 (2.5%)	0.752
Coarctation of Aorta	2 (1.8%)	18 (0.6%)	10 (0.7%)	0.783
AAA	11 (10.1%)	212 (7.1%)	101 (7.0%)	0.470
Renal Cyst	6 (5.5%)	101 (3.4%)	38 (2.6%)	0.162
Liver Cyst	2 (1.8%)	18 (0.6%)	5 (0.3%)	0.090
History of Steroid Use	0 (0%)	69 (2.3%)	34 (4.9%)	0.307
Chronic Kidney Disease	1 (0.9%)	143 (4.8%)	71 (4.9%)	0.141

**Table 4:** Summary of the whole exome sequencing (WES) results among the 3746 patients comprising Yale’s Aortic Institute Database. Bold text represets categories, while non-bold text represents sub-categories. Statistical analysis utilizing a Pearson’s chi-squared test. Significant *p*-value < 0.001 (*). BAV: bicuspid aortic valve, TAAD: thoracic aortic aneurysm and dissection, WES: whole exome sequencing, VUS: variant of uncertain significance.

Category	*n* (%)
**Patients with Whole Exome Sequencing (WES)**	**631 (16.8%)**
**Connective Tissue Status Among WES Patients**	
Syndromic	25 (4.0%)
Non-syndromic	552 (87.5%)
Unknown	54 (8.6%)
**Family History Among WES Patients**	
Proven Famliy History	179 (28.4%)
No Family History	263 (41.7%)
**Patients with Positive WES results**	**141 (22.3%)**
Familial TAAD Status Among Positive WES Patients *	
Familial TAAD Patients with Positive WES	70 (49.6%)
Non-Familial TAAD Patients with Positive WES	26 (18.4%)
Connective Tissue Status Among Positive WES Patients *	
Syndromic	12 (8.5%)
Non-syndromic	103 (73.0%)
Unknown Connective tissue status	26 (18.4%)
**Acute Aortic Dissection Among Positive WES Patients**	14 (9.9%)
**BAV Status Among Positive WES Patients**	72 (51.1%)
**Total Genetic Variants Identified**	**170**
**Classification of Genetic Variants**	
Pathogenic	13 (7.6%)
Likely Pathogenic	16 (9.4%)
Variants of Uncertain Signifcance (VUS)	134 (78.8%)
Not classified	7 (4.1%)

**Table 5:** Whole exome sequencing (WES) results of 631 patients. WES results were considered positive when at least one pathogenic, likely pathogenic variant, or variant of uncertain significance (VUS) was found. Bold text represents headings. Statistical analysis utilizing a Pearson’s chi-squared test. Significant *p*-value < 0.001 (*).

**Disease Causing or Ssuspicious Variants (*n* = 170)**			
Pathogenic	Likely pathogenic	Variants of Uncertain Significance (VUS)	Unclassified
13 (7.6%)	16 (9.4%)	134 (78.8%)	7 (4.1%)
**Disease Causing or Suspicious Variants (positive WES results)**			
Familial patients		70 *	
Non-familial patients		26 *	

## Data Availability

The data presented in this study are available on request from the corresponding author due to patient privacy restrictions.

## Supplementary Materials

The following supporting information can be downloaded at: https://www.mdpi.com/article/10.3390/genes17101205/s1, Figure S1: Skewness Analysis—Age Distribution; Figure S2: Familial TAAD Pedigrees. Figure S1. Analysis of skewness of the age distribution in the syndromic, non-syndromic, and sporadic groups. Analysis and plotting was performed using R 4.4.2 (R Foundation for Statistical Computing, Vienna, Austria). Figure S2. Additional non-syndromic thoracic aortic aneurysm family pedigrees from the Yale’s Aortic Institute database. Squares represent men, circles represent women, diamond shapes represent unknown gender. Blackened squares or circles represent affected patients with aortic aneurysms, gray squares or circles represent unknown disease status. Crossed out squares or circles represent deceased individuals. Visualization of pedigrees was done using QuickPed 4.5.0 (Oslo, Norway) [24].

## Abbreviations

CT	Computed tomography
MRI	Magnetic resonance imaging
TAA	Thoracic aortic aneurysm
TAAD	Thoracic aortic aneurysm and dissection
TEE	Transesophageal echocardiography
TTE	Transthoracic echocardiography
VUS	Variant of uncertain significance
WES	Whole exome sequencing
